# Enhanced DNA Damages of Human Prostate Cancer Cells Induced by Radiofrequency Capacitive Hyperthermia Pre- and Post X-rays: 6 MV versus 15 MV 

**DOI:** 10.22074/cellj.2017.4749

**Published:** 2017-05-17

**Authors:** Seied Rabi Mahdavi, Azam Janati Esfahani, Mohammad Bagher Shiran, Samideh Khoei, Nader Estiri

**Affiliations:** 1 Radiation Biology Research Center, Department of Medical Physics, School of Medicine, Iran University of Medical Sciences, Tehran, Iran; 2 Department of Medical Physics, School of Medicine, Iran University of Medical Sciences, Tehran, Iran; 3 Department of Engineering, Science and Research Branch, Islamic Azad University, Tehran, Iran

**Keywords:** Prostate Cancer, Comet Assay, Hyperthermia, Radiation, Spheroid

## Abstract

**Objective:**

This study aimed to determine the effect of 13.56 MHz radiofrequency (RF) capacitive hyperthermia (HT) on radiosensivity of human prostate cancer cells pre and post X-ray radiation treatment (RT).

**Materials and Methods:**

In this experimental study, the human prostate cancer cell line DU145 was cultured as 300 µm diameter spheroids. We divided the spheroids into group I: control, group II: HT at 43˚C for 30 minutes (HT), group III: 4 Gy irradiation with 6 MV X-ray [RT (6 MV)], group IV: 4 Gy irradiation with 15 MV X-ray [RT (15 MV)], group V: HT+RT (6 MV), group VI: HT+RT (15 MV), group VII: RT (6 MV)+HT, and group VIII: RT (15 MV)+HT. The alkaline comet assay was used to assess DNA damages in terms of tail moment (TM). Thermal enhancement factor (TEF) was obtained for the different treatment combinations.

**Results:**

Mean TM increased with increasing photon energy. Group II had significantly greater TM compared to group I. Groups III and IV also had significantly higher TM compared to group I. Significant differences in TM existed between groups V, VII, and III (P<0.05). We observed significant differences in TM between groups VI, VIII, and IV. TEF values demonstrated that enhanced response to radiation was more pronounced in group V compared to the other combined treatments.

**Conclusion:**

Our results suggest that HT applied before RT leads to higher radiosensivity compared to after RT. HT at 43˚C for 30 minutes added to 6 MV X-ray causes higher enhancement of radiation compared to 15 MV X-ray.

## Introduction

Hyperthermia (HT), at a temperature range of 42-45˚C, is a potent radiosensitizer that can cause irreversible damage due to protein degradation and the lack of DNA double-strand break (DSB) repair in cells (1,2). Increasing use of HT has led to a need to develop new devices that treat deep seated tumors such as prostate cancer. However, HT is limited in clinical therapy because of the difficulties to target this approach to the tumor volume. Radiofrequency (RF) capacitive HT combined with radiotherapy (RT) is recommended as treatment for prostate cancer. In the 13.56 MHz capacitive coupling electro HT system, a part of the patient’s body is placed as the dielectric material between both electrodes of the device. Consequently, heat is generated in the tissue (3). Cancer tissue has certain physical properties that Which makes its temperature goes higher than normal tissue (4); actually, autofocusing occurs. External radiation therapy is usually applied with high-energy photons in the range of 6-18 MV. High energy photons (15-18 MV) acquire deeper penetration and better dose distribution in prostate tissue. In the last few years the use of high daily dose radiotherapy techniques have been increasingly used for prostate cancer treatment (5-7). Ionizing radiation can lead to DNA damages in exposed tissue, which may lead to loss of clonogenic survival of tumor cells that is the main goal of radiotherapy. DSBs are assumed to be the major radio-toxic damage after radiotherapy treatment of cancer (8). The addition of heat to radiation, by inhibiting DSB repair, intensifies the toxic effects of radiation. The additive effect of HT combined with radiation treatment can be estimated by the thermal enhancement factor (TEF) defined as the response after radiation with heat divided by the response after radiation alone at the same radiation dose. 

Despite numerous researches, it is not known if HT before or after irradiation can enhance radiation damage. The effect of combined HT and radiation in prostate cancer cells has been researched (9,10). However, there are no comparative investigations of sequential treatment schedules in prostate cancer. In this study, we have sought to assess the influence of sequence between RF capacitive HT on radiosensitivity of human prostate cancer cells pre and post X-ray (6 and 15 MV). 

## Materials and Methods

### Cell line

In this experimental study, we purchased the human prostate carcinoma cell line DU145 from Pasteur Institute of Iran. This cell line was cultured in Roswell Park Memorial Institute medium (RPMI-1640, Gibco, NY, USA) supplemented with 10% heat-activated fetal bovine serum (FBS, Gibco, NY, USA), 100 U/ml of penicillin and 100 mg/ml of streptomycin (Biowest, Nuaille, France). 

### Monolayer culture and doubling time calculation

DU145 cells were cultured as a monolayer at a density of 10^4^ cells/cm^2^ in T-25 tissue culture flasks (Nunc, Roskilde, Denmark). The cultures were maintained at 37˚C in a humidified atmosphere of 5% CO_2_ . Cells were propagated by trypsinizing cultures with 1 mM EDTA/0.25% w/v Trypsin (Sigma- Aldrich, MO, USA) in phosphate-buffered saline (PBS, SPL Life Sciences Co., Ltd., Korea). 

After three passages, 2×10^4^ cells were cultured per well in 24-well plates (SPL). At 24-hour intervals, the cells from three wells were removed by 1 mM EDTA/0.25% Trypsin (w/v) and counted in a hemocytometer. An average of the cell counts was used to determine growth doubling time, which was achieved at 36 hours. We calculated growth doubling time using the slope of the logarithmic phase of the growth curve. 

### Spheroid culture

**Fig.1:**
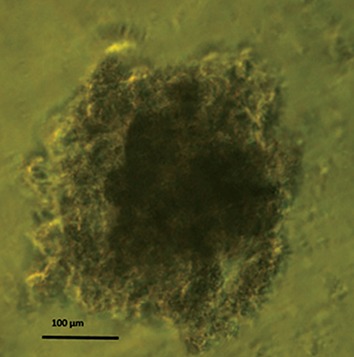
Phase contrast micrograph of DU145 spheroids (300 μm diameter, magnification: ×10).

We used the liquid overlay technique to establish spheroids (11). A total of 5×10^5^ cells were seeded in 100 mm Petri dishes (Jet Biofil, Co., Ltd., China) coated with a thin layer of 1% agar (Sigma- Aldrich, MO, USA) with 10 ml of RPMI 1640 supplemented with 10% FBS. The plates were incubated at 37˚C in a humidified atmosphere of 5% CO_2_ . Approximately 5 ml of culture medium was replaced by fresh medium twice per week. Spheroids grew exponentially with an apparent volume doubling time of 106.56 hours. After approximately 20 days, when spheroids reached 300 µm in diameter (Fig.1), they were transferred to sterile T-12.5 flasks (Jet Biofil, Co., Ltd., China) filled with RPMI 1640 medium for subsequent exposure to radiation, heat, or their combination. 

### Radiation treatment

The 300 µm diameter spheroids were transferred to T-12.5 flasks (Jet Biofil, Co., Ltd., China), completely covered by RPMI1640 medium, and sealed. For all irradiation, we placed the flasks in the center of a water phantom that had dimensions of 30×30×15 cm. Cells were exposed to a 4 Gy dose of either 6 or 15 MV X-ray by a Siemens Primus linear accelerator. The control sample received no radiation exposure. 

### Hyperthermia

HT treatments at 43˚C for 30 minutes were delivered by a RF capacitive HT system, Celsius TCS (Celsius42+GmbH Company, Cologne, Germany), at a frequency of 13.56 MHz. Both electrodes of the device were 25 cm in diameter. A T-12.5 flask that contained the spheroids and RPMI1640 was placed between two electrodes of the device. We used a specific treatment protocol to generate HT for 30 minutes at 43˚C ± 0.1. Cells exposed to 37˚C served as the control sample. 

### Combined treatment of hyperthermia and radiation

Irradiation was performed at a dose of 4 Gy by either 6 or 15 MV X-ray after or before HT of 30 minutes. The interval between the end of HT and the beginning of radiation exposure was fixed at 15 minutes. The radiosensitizing effect of heat was expressed as TEF, which is the ratio of the response of cells to HT+RT or RT+HT divided by RT alone at the same radiation dose. 

### Trypan blue exclusion assay

Treated cells were separated and mixed with trypan blue at a 9:1 ratio. After 3-5 minutes, this solution was observed under a light microscope (Bell, INV-100-FL). Blue-colored cells were considered nonviable. The ratio of unstained cells to total number of cells was reported as the percentage of viability for each sample. 

### Alkaline comet assay

We used the alkaline comet assay to assess for the presence of DNA damage induced by each treatment. This method was previously established by Fazeli et al. (12). By measuring the fluorescence intensity using the CometScore software, DNA damages were quantified as an increase in tail moment (TM), as the product of the amount of DNA (fluorescence) in the tail and the distance between the means of the head and tail fluorescence distributions. 

### Statistical analysis

Each experiment was performed in triplicate. Data presented on the curves were expressed as mean ± SEM and other data were expressed as the mean ± SD. Statistical analysis was performed using one-way analysis of variance (ANOVA) followed by Tukey’s test as the post-hoc analysis using SPSS version 17. A P<0.05 was considered statistically significant. 

## Results

### The effect of treatments on cell viability

**Fig.2:**
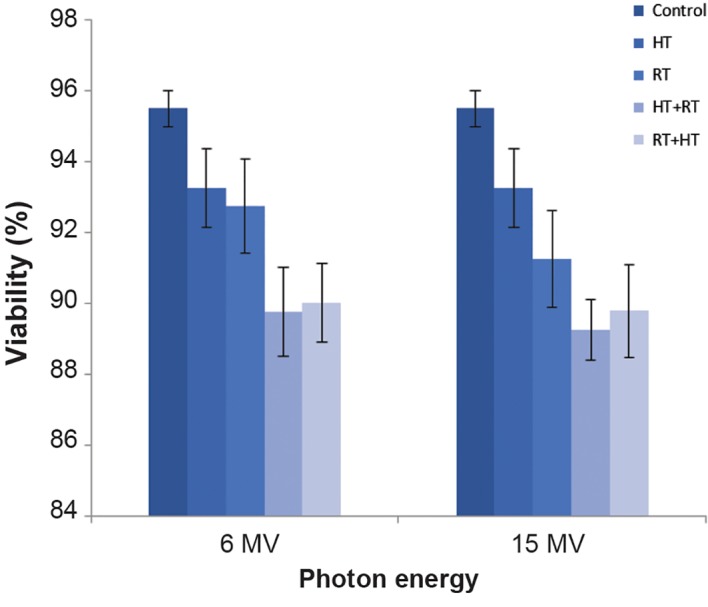
The effect of radiation treatment (RT) 6 MV and 15 MV, hyperthermia (HT), and their combination on viability of DU 145 cells in a spheroid culture. About 1 hour after treatment we assayed cell viability by the trypan blue dye exclusion test as previously described (mean ± SEM of three experiments).

The treatments had no significant effects on cell viability. Figure 2 shows the fraction of viability after each treatment. Trypan blue exclusion staining showed that the strongest reduction in viable cell numbers in the HT+RT (15MV) group, which was 89.25 ± 2.2%. 

### The effect of treatments on DNA damages assayed by alkaline comet assay 

**Fig.3:**
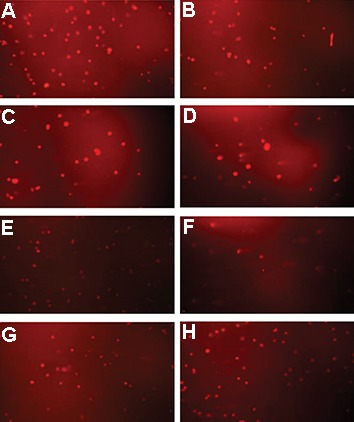
Microphotography of alkaline comet assay of 300 μm DU145 spheroids by fluorescence microscopy. A. Control group, B. Hyperthermia (HT), C. Radiation treatment [RT (6 MV)], D. RT (15 MV), E. HT+RT (6 MV), F. HT+RT (15 MV), G. RT (6 MV)+HT, and H. RT (15 MV)+HT.

Figure 3 shows the microphotography of the comet assay of DU145 cells in the control, HT, RT, HT+RT, RT+HT treatments for 6 MV and 15 MV photon energies. Damaged cells can be seen with tails in Figure 3. A bright head of undamaged DNA with a comet’s tail was drawn outward. Damaged DNA strands are in the tail. 

**Fig.4:**
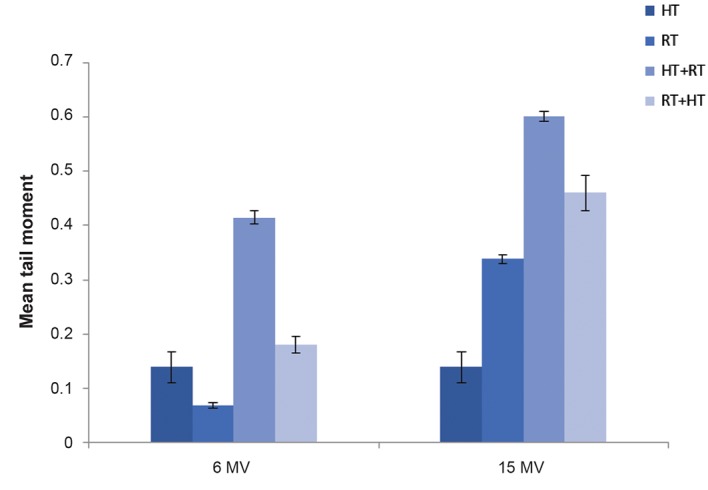
The effect of radiation treatment (RT) and hyperthermia (HT) on DNA damages of DU 145 cells from spheroid culture is depicted. Cells were exposed to 4 Gy dose of either 6 MV or 15 MV photons. RT combined with 30 minutes radiofrequency (RF) HT at 43˚C. Tail moment induced by HT, RT, HT+RT and RT+HT normalized to the control value (mean ± SEM of three experiments).

The mean TM obtained for each treatment was normalized to the control value and reported as an indication of DNA damage. Our results (Fig.4) showed a significant difference in TM in the groups that received RT compared with the control group. The increase in DNA damage by the addition of HT significantly increased compared to cells that received RT alone (P<0.05). The TM in the HT alone group showed a significant difference compared with the control group. The data indicated that DNA damages increased with increased photon energy. Furthermore shows a higher DNA damages for HT+RT than the opposite sequence (RT+HT). Figure 4 shows that the TM had a maximum increase in the cells treated with HT+RT (15 MV). 

A method of examining the radiosensitizing effect of HT is to compare the levels of TM achieved for the addition of HT to radiation to a given radiation dose alone. The ratio of two TM was introduced in term of TEF. Table 1 shows that RT (6 MV)+HT had a TEF of 2.59 and RT (15 MV)+HT had a TEF of 1.36. HT+RT (6 MV) had a TEF of 6, whereas HT+RT (15 MV) had a TEF of 1.78. The heating effect of RF HT was more prominent after irradiation with 6 MV. 

**Table 1:** Radiosensivity effect of RF HT measured by TEF. The effect of changing the sequence of two treatments is shown


Photon energy	Mean TM^* ^RT alone	Mean TMRT+HT	TEF^**^	Mean TM HT+RT	TEF

6 MV	0.069 ± 0.008^***^	0.179 ± 0.026	2.59	0.415 ± 0.022	6
15 MV	0.338 ± 0.013	0.460 ± 0.056	1.36	0.602 ± 0.016	1.78


*; Mean TM values in this table were obtained from data shown in Figure 4, **; Mean TM of cells after RT combined with HT was divided by TM after RT alone, ***; Mean TM ± SD, RF; Radiofrequency, HT; Hyperthermia, RT; Radiation treatment, TEF; Thermal enhancement factor, and TM; Tail moment.

## Discussion

HT is a cancer treatment modality that increases the body’s normal temperature to 42-45˚C. HT has the capability to sensitize cells to radiotherapy/chemotherapy (13). HT, as an adjunct to ionizing radiation, has been used to treat prostate cancer (10). DU145, a prostate cancer cell line, has the capability to generate large, well-balanced spheroids in the liquid overlay culture technique (14). Cells in the spheroid structure represent a three-dimentional model, which is the same as the tumor structure. The 300 μm spheroids have a large hypoxic area. HT at 43˚C alone leads to hypoxic cell death, mainly through the induction of lethal protein denaturation (2, 15, 16). It has been accepted that HT at this temperature inhibits DNA repair (2). Oei et al. (17) and Roti Roti (18) have claimed that HT may induce DNA fragmentation and chromosomal aberrations, either by causing protein degradation or by interfering with replication and may lead to cell death, either directly or by induction of apoptosis. Though heat is not able to cause serious DNA damage, it has the potential to denature DNA repair enzymes. Therefore in conjunction with radiation, heat can enhance DNA damage (2).

Radiobiologically, prostate cancer has a low alpha/beta ratio. Hence, hypofractionation where large doses are delivered in a few fractions, has been utilized in external beam irradiation of prostate cancer in the last few years (19). In this study, we have examined the effect of adding 4 Gy as a large dose of RT to HT. Our ultimate goal was to find a good regimen of combined heat and radiation that would achieve desirable treatment for prostate cancer cells. In the current study, we evaluated the effect of the sequence of heat and radiation in combined RF HT and photon irradiation. We compared the radiosensitizing effect of RF HT (30 minutes at 43˚C) administered before and after 6 MV and 15 MV X-rays RT. The viability assay showed no significant effects on cell viability in the different treatments compared to the control. All treatments resulted in greater than 89% viability. Hence, we concluded that no treatments immediately led to cell death. We observed increased TM with increased photon energy in both the radiation alone treatments and those combined with HT. 

Through the interaction of high energy photons (>7 MV) with high Z materials such as the linear accelerator head, neutrons contaminate the photon beam (20). Some researchers have measured the amount of neutron energy created from 15 MV X-rays that originated from a clinical linear accelerator (21,22). The radiation weighting factor of this neutron energy range is approximately 15 (23), therefore neutrons have a much larger relative biological effectiveness (RBE) compared to photons and result in increased biological damage (24,25). According to Kry et al. (26), 6 MV X-rays do not create any photoneutrons. As a result, the higher DNA damages that have resulted from 15 MV compared with 6 MV photon energy can be attributed to neutron contamination produced by high-energy photons. Our data demonstrated that HT+RT (15 MV) treatment would have a greater effect on DNA damage to DU145 cells than other treatments. The data for TEF show that DU145, the human prostate carcinoma cell line, has been radiosensitized by RF HT and heating before radiation resulted in a higher TEF compared to RT before HT. The data in the present study agreed with the results observed in previous studies that used other types of HT (27,28). 

Researches have shown that the non-thermal effect of RF exposure does not cause DNA bond breakage (29,30). According to these findings, it can be determined that the enhanced DNA damages in HT and combination therapy is because of the effect of heat generated from the RF capacitive device. In the combination therapy, we took more radiosensitivity in HT+RT than reverse sequence. Repair of hyperthermic damage requires more time than repair for a similar radiation damage (31,32). In this sequence in which HT was exposed before RT, loss of DSB repair has led to radiosensitization. Furthermore, HT in this situation acted as a radiosensitizer. The late damage would probably be enhanced since HT can act as a "high dose per fraction" and thus enhance late radiation damage (33). 

Researchers have reported some physical and biological factors that affect the degree of heat radiosentization, which include "cell line, thermotolerance, recovery and repair, the phase of the cell cycle, temperature, heating time, treatment sequence and radiation dose" (27,34). However, the main factor of heat radiosensitization is the sequence and interval between application of the two modalities (27) Our results are in good agreement with these results. 

Our findings showed that the TEF data from HT+RT and RT+HT treatments reduced with increasing photon energies. Two theories can be proposed. Firstly, hyperthermia alone, about DU145 cells, only begins to enhance the radiation response. This enhancement increased with reduced photon energy. Secondly, 15 MV photons caused more severe DNA damage compared to 6 MV. Hence, after the addition of HT to RT, other injuries than DNA damages might be induced which were not detectable by the alkaline comet assay. 

## Conclusion

Our results showed that HT+RT could radiosensitize more prostate cancer cells compared to RT+HT. We demonstrated that the radiosensitization effect of combined treatments of heat and radiation exposed to lower photon energy were higher than in which cells had exposure to higher energy at the same absorbed dose. We reported another factor that affected the degree of radiosensivity as "photon energy".We recommend that further investigations should elucidate the role of photon energy in complex mechanisms of thermo-radiosensitization of human prostate cancer cells. 
